# Passion fruit juice with different sweeteners: sensory profile by descriptive analysis and acceptance

**DOI:** 10.1002/fsn3.195

**Published:** 2015-01-06

**Authors:** Izabela Furtado de Oliveira Rocha, Helena Maria André Bolini

**Affiliations:** Faculty of Food Engineering, Food and Nutrition Department, University of CampinasR. Monteiro Lobato 80, 6121, Campinas, Brazil

**Keywords:** Acceptance, passion fruit juice, PLS, QDA, sensory analysis, sweeteners

## Abstract

This study evaluated the effect of different sweeteners on the sensory profile, acceptance, and drivers of preference of passion fruit juice samples sweetened with sucrose, aspartame, sucralose, stevia, cyclamate/saccharin blend 2:1, and neotame. Sensory profiling was performed by 12 trained assessors using quantitative descriptive analysis (QDA). Acceptance tests (appearance, aroma, flavor, texture and overall impression) were performed with 124 consumers of tropical fruit juice. Samples with sucrose, aspartame and sucralose showed similar sensory profile (*P* < 0.05), without bitter taste, bitter aftertaste, and metallic taste, and samples with sucrose and sucralose did not differ from each other for the attribute sweet aftertaste. Passion fruit flavor affected positively and sweet aftertaste affected negatively the acceptance of the samples. Samples sweetened with aspartame, sucralose, and sucrose presented higher acceptance scores for the attributes flavor, texture, and overall impression, with no significant (*P* < 0.05) differences between them. Aspartame and sucralose can be good substitutes for sucrose in passion fruit juice.

## Introduction

Passion fruit is one of the most popular tropical fruits having a floral, estery aroma with an exotic tropical sulfury note. The yellow passion fruit, one important commercial variety, is more acidic and mainly used for juice preparation (Deliza et al. 2005). In addition, the production of concentrated passion fruit juice increased from approximately 4400 tons in 2005 to approximately 11,200 tons in 2010 in Brazil (IBGE 2010).

Parallel to the production and consume of concentrated passion fruit juice, nowadays, sweeteners have been used in foods, driven primarily by consumer demand for foods with lower carbohydrate content and energy density compared to sugar-containing variants. Sweeteners and products formulated with sucrose replacers increase consumer choice by providing the potential to reduce calories and to enhance nutritional and health benefits. Thereby, the availability and acceptability of the passion fruit juice in the Brazilian market and the increasing demand for low-calorie and low-sugar products should be evaluated together (De Marchi et al. 2009, Shortt 2014).

This way, sweeteners are added to foods to replace the sweetness normally provided by sucrose without contributing significantly to available energy and are a means for consumers to control caloric or carbohydrate intake (Pinheiro et al. 2005; Trevisam Moraes and Bolini 2010; O'Mullane et al. 2014). Several sweeteners are permitted for use in diet foods and beverages, which should have low caloric density on a sweetness equivalency basis, be physiologically inert, organoleptically acceptable, commercially viable, besides assisting in weight-loss maintenance and diabetes management, and dental cavities prevention (Malik et al. 2002).

The sweeteners must be studied in low-sugar products, because that sensory characteristics, acceptance and preference of low-calorie food products are highly dispersion matrix dependents. So, it is essential to study the substitution of sucrose by high-intensity sweeteners every time a formulation or concentration are changed or a new product is developed (De Marchi, et al. 2009; Pinheiro et al. 2005). And the sweeteners are only successful if they show a perfect match with the sensory profile of sucrose (Portmann and Kilcast 1996; Cadena and Bolini 2012).

Therefore, sensory evaluation is essential for its implementation in juice and blend formulations, and for the consumption of passion fruit, once the juice has intense acidic flavor, therefore water, sugar, or high-intensity sweeteners should be added to provide a palatable juice (Deliza et al. 2005).

The objective of this study was to evaluate the effect of different sweeteners on the sensory profile, acceptance, and drivers of preference of passion fruit juice samples.

## Materials and Methods

Passion fruit juice samples were prepared with unsweetened concentrated juice (Da Fruta®, Araguari, Minas Gerais, Brazil). The samples were sweetened with sucrose and five different sweeteners: aspartame (Ajinomoto, São Paulo, São Paulo, Brazil), stevia extract, sucralose and neotame (Tovani-Benzaquen, São Paulo, Brazil), cyclamate and saccharin (Sweet Mix, Brazil).

### Physicochemical analyses

In this study, we evaluated color, pH, and soluble solid. Sample color (L*, a*, b*) was determined in a Hunterlab Colorquest II model colorimeter (Reston, Virginia, USA). The apparatus was calibrated with the D65 illuminant (6900K), the reading being carried out using a 10 mm quartz cuvette, illuminant C and hue of 10°, with Regular Transmission (RTRAN) at the moment of reading and a white reference plate (C6299 Hunter Color Standard). The pH of the samples was determined using an Orion Expandable Ion Analyzer EA 940 pH meter. The total titratable acidity was measured using AOAC (1997) and expressed as % citric acid. The percentage of soluble solids in terms of °Brix was determined using a Carl Zeiss 844976 Jena refractometer with AOAC (1997). And finally, the ratio was calculated as the ratio of total soluble solids (°Brix) to titratable acidity (Sabato et al. 2009).

### Descriptive analysis

Approval for the study was obtained from the Ethics Committee of the University of Campinas, and written consent was given by all volunteers.

The sensory profiles were generated by a panel of 12 trained judges between 18 and 35 years of age, undergraduate or postgraduate students and employees, from the University of Campinas, Brazil, who were experienced in food and beverage sensory evaluation using quantitative descriptive analysis (QDA, Stone et al. 1974; Meilgaard et al. 1999; Stone and Sidel 2004). The panelists were initially screened using the sequential method proposed by WALD (Amerine et al.^1965^), in which triangle tests are used to select subjects with a good ability to discriminate samples. A series of triangular tests was conducted, in which the candidates were offered two passion fruit juice samples: A (containing 35 g/L sucrose), and B (containing 50 g/L sucrose), with significant difference of 0.1%.

The parameters used in the sequential analysis were: p0 = 0.45 (maximum unacceptable ability), p1 = 0.75 (minimum acceptable ability), a = 0.10 (likelihood of accepting a candidate without sensory acuity) and b = 0.10 (likelihood of rejecting a candidate with sensory acuity). Based on these parameters, the sensory panelists were selected according to the number of triangular tests and the cumulative number of correct judgments.

#### Equi-sweetness determination

Initially, a study to determine the ideal sweetness of the passion fruit juice samples sweetened with sucrose was carried out. An acceptance test using a Just About Right (JAR) scale (Meilgaard et al. 2004) was performed with 60 consumers of tropical fruit juices. The samples were sweetened with sucrose at five concentrations: 5.0, 7.5, 10.0, 12.5, and 15.0 g/100 g, in order to determine the ideal sweetness according to consumer's acceptance.

After the determination of ideal sweetness, the relative sweetness of the sweeteners was measured using the Magnitude Estimation method (Stone and Oliver 1969), which makes possible a direct quantitative measurement of the subjective intensity of sweetness.

Five concentrations of each sweetener were evaluated. Firstly, the passion fruit juice sample sweetened with sucrose in the ideal concentration (reference sample) was presented, followed by the samples containing five different concentrations of each sweetener, through randomized complete sets. The subjects were served 30 mL of each sample, and 90 mL of reference sample. Each sweetener was tested in different days. Water was provided for palate cleansing.

The reference sample was taken as intensity of 100, followed by a random series of samples with intensities both less and greater than the reference. The subject was asked to estimate the sweetness intensity of the unknown samples in relation to the reference. For example, if the sample is two times sweeter than the reference, it should receive an intensity of 200, if the sample is half as sweet, the intensity should be 50, and so on. Assessors were instructed not to rate the samples' intensity as zero.

The “ideal sweetness determination” and “equi-sweetness determination” were described according to Rocha and Bolini (2014), and all juices were prepared to be equi-sweet. The juices with different sweeteners developed in this study are presented in Table1.

**Table 1:** Equi-sweet concentration of the sweeteners used this study

Sweeters	Concentration equivalent (g/100 mL)
Sucrose	9.400
Aspartame	0.05477
Sucralose	0.01593
Stevia	0.09924
Cyclamate/saccharin blend 2:1	0.03584
Neotame	0.00156

#### Training and selection of panelist

Using Kelly's Repertory Grid Method described by Moskowitz (1983), panelists evaluated the samples of passion fruit juice with five different sweeteners (sucrose, aspartame, sucralose, stevia, cyclamate/saccharin blend 2:1, and neotame). The individuals received two passion fruit juice samples and individually described their similarities and differences with respect to appearance, aroma, flavor, and texture. As a group, the panelists then discussed the terms generated by each individual and, with the supervision of a panel leader, consensually defined the terms that adequately described appearance, aroma, flavor and texture similarities and differences amongst the samples, writing down their definitions and suggesting references for training purposes. In subsequent sessions, the suggested references were presented, discussed, and approved or modified by the group. During this process, eighteen sensory descriptors were consensually generated, as well as the written definitions and references for each one (Table2).

**Table 2:** Attributes and reference standards generated by the sensory panel

Attributes	Definitions	References
Appearance		
Yellow color	Yellowish orange color characteristic of passion fruit juice	Weak: passion fruit juice concentrate (Maguary®) – 1 part pulp/20 parts water
		Strong: passion fruit juice concentrate (Maguary®) – 1 part pulp/1 part water
Apparent viscosity	Flow rate of juice in the cup wall	Weak: passion fruit juice concentrate (Maguary®) – 1 part pulp/20 parts water
		Strong: passion fruit juice concentrate (Maguary®) – 1 part pulp/1 part water
Brightness	The degree to which the sample reflects light in one direction	Weak: cooked egg yolk
		Strong: peach gelatin (Dr Oetker®) – prepared according to manufacturer
Aroma		
Passion fruit	Characteristic aroma from natural passion fruit juice	Weak: passion fruit juice concentrate (Maguary®) – 1 part pulp/20 parts water
		Strong: passion fruit juice concentrate (Maguary®) – 1 part pulp/1 part water
Sweet	Aroma due to the presence of sucrose and other sugar from passion fruit	Weak: passion fruit juice concentrate (Maguary®) – 1 part pulp/6 parts water + 5 g loaf sugar (Caravelas®)
		Strong: loaf sugar (Caravelas®)
Acid	Aroma related to the presence of characteristic organic acids from passion fruit	Weak: passion fruit juice concentrate (Maguary®) – 1 part pulp/20 parts water
		Strong: passion fruit juice concentrate (Maguary®) – 1 part pulp/2 part water
Cooked	Characteristic aroma from passion fruit submitted to thermal processing (heat)	Weak: passion fruit juice concentrate (Maguary®) – 1 part pulp/20 parts water
		Strong: pulp of passion fruit juice (DeMarchi®)
Flavor		
Passion Fruit	Characteristic flavor from natural passion fruit juice	Weak: passion fruit juice concentrate (Maguary®) – 1 part pulp/20 parts water
		Strong: passion fruit juice concentrate (Maguary®) – 1 part pulp/1 part water
Cooked	Characteristic flavor from passion fruit submitted to thermal processing (heat)	Weak: passion fruit juice concentrate (Maguary®) – 1 part pulp/20 parts water
		Strong: pulp of passion fruit juice (DeMarchi®)
Sweet taste	Taste stimulated by the presence of sucrose and other substances, such as sweetner	Weak: passion fruit juice concentrate (Maguary®) – 1 part pulp/6 parts water + 5 g/L loaf sugar (Caravelas®)
		Strong: passion fruit juice concentrate (Maguary®) – 1 part pulp/6 parts water + 20 g loaf sugar (Caravelas®)
Bitter taste	Characteristic taste from caffeine	Weak: passion fruit juice concentrate (Maguary®) – 1 part pulp/20 parts water
		Strong: passion fruit juice concentrate (Maguary®) – 1 part pulp/6 parts water + 1 g/L cafeine
Sweet aftertaste	Sweet sensation perceived at the back of the throat after swallowing.	Weak: passion fruit juice concentrate (Maguary®) – 1 part pulp/6 parts water
		Strong: passion fruit juice concentrate (Maguary®) – 1 part pulp/6 parts water + 15 g/L aspartame (Ajinomoto®)
Bitter aftertaste	Bitter sensation perceived at the back of the throat after swallowing	Weak: passion fruit juice concentrate (Maguary®) – 1 part pulp/6 parts water
		Strong: passion fruit juice concentrate (Maguary®) – 1 part pulp/6 parts water + 2 g/L stevia extrat (Tovani-Benzaquen®)
Sour taste	Taste related to the presence of characteristic organic acids from passion fruit	Weak: passion fruit juice concentrate (Maguary®) – 1 part pulp/20 parts water
		Strong: passion fruit juice concentrate (Maguary®) – 1 part pulp/2 parts water
Sour aftertaste	Sour sensation perceived at the back of the throat after swallowing	Weak: passion fruit juice concentrate (Maguary®) – 1 part pulp/20 parts water
		Strong: passion fruit juice concentrate (Maguary®) – 1 part pulp/2 parts water
Adstringency	Harsh sensation perceived in mouth and tongue characteristic of passion fruit	Weak: passion fruit juice concentrate (Maguary®) – 1 part pulp/20 parts water
		Strong: cashew juice concentrate (Maguary®)
Metallic	Flavor associated with “rust”/”metal”	Weak: passion fruit juice concentrate (Maguary®) – 1 part pulp/6 parts water
		Strong: passion fruit juice concentrate (Maguary®) – 1 part pulp/6 parts water + 0.5 g/L FeSO_4_
Texture		
Viscosity	Perceived time during swallowing	Weak: passion fruit juice concentrate (Maguary®) – 1 part pulp/20 parts water
		Strong: cashew juice concentrate (Maguary®)

In consensus, the panelists also elaborated a sensory descriptive term (Table2) for the samples, associating each descriptor with a 9-cm unstructured scale, anchored at its left and right extremes by the terms “none/weak” and “strong”, respectively.

After a training period, a final selection of the panelists was carried out, where each one evaluated three fruit juice samples with three replications. Analysis of variance (ANOVA) with sample (pF_sample_) and replication (pF_replication_) as source of variation was carried out for each panelist and each sample.

The level of significance for the source of variation “sample” (pF_sample_ ≤ 0.50) was used as the criterion to estimate the discriminative power of each judge, and the level of significance for the source of variation “replication” (pF_replication_ ≥ 0.05) was used as the criterion to estimate the reproducibility of each judge. Only individuals showing adequate discriminative power (pF_sample_ ≤ 0.50), reproducibility (pF_replication_ ≥ 0.05), and consensus with the rest of the panel for at least 60% of the descriptors were selected to take part in the descriptive panel (Damásio and Costell 1991).

#### Sensory profile

Samples of passion fruit juice (30 mL) were presented in codified white disposable cups with 3 digits, and sensory analyses were carried out in individual air-conditioned (22°C) booths with white light.

The samples were tested in complete balanced block design with tree repetitions, and the order of presentation of the samples was balanced for the first-order effect (MacFie et al. 1989).

### Acceptance test

One hundred and twenty-four consumers of the tropical fruit juice evaluated all the six passion fruit juice samples to determine liking of appearance, aroma, flavor, texture, and overall impression.

Acceptance was determined using a 9-cm linear hedonic scale (not structured) (Stone and Sidel 2004), with anchors of “dislike extremely” on the left and “like extremely” on the right.

All samples were presented using a balanced complete block design (MacFie et al. 1989). According to Greene et al. (2006), sensitivity in defining consumer perception is greater with the use of line scales than with the 9-point hedonic scale.

The sensory profile results were performed using the SAS version 9.1.3 (SAS_ Institute, Cary, NC). The sensory descriptive data were evaluated by ANOVA (sources of variation: passion fruit juice, judges, passion fruit juice_judge) followed by Tukey's test for multiple mean comparisons (*P* < 0.05). The sensory and analytical data were also analyzed by Principal Component Analysis (PCA), and correlated with the analytical data using the Pearson correlation coefficient.

The acceptance results were analyzed by ANOVA, using two factors (consumer and passion fruit juice), and Tukey's test. Descriptive information obtained from the taste panel was related to the consumer preference data using partial least squares (PLS) regression (Melo et al. 2009; Bayarri et al. 2012; Cadena et al. 2012). PLS regression involved the development of a matrix data where the lines were the passion fruit juice samples (6 lines), and the columns were the eighteen attributes used by the consumers to describe the samples. Statistical analyses were carried out using XLSTAT for Windows version 2012.5 (Addinsoft, Paris, France) at a 5% significance level.

## Result and Discussion

### Physicochemical analyses

With respect to the pH values of the passion fruit juice (Table3), there were no significant differences (*P* > 0.05) between the different samples. There was a significant difference (*P* > 0.05) in the titratable acidity values, and the sample with sucrose presented the lowest mean.

**Table 3:** Physicochemical characteristics of passion fruit juice sample

	pH	Titratable acidity (%)	°Brix	Ratio^1^	L*	a*	b*
Sucrose	2.82^a^	0.4621^c^	10.00^a^	21.64	45.6867^a^	6.1567^a^	30.5600^a^
Aspartame	2.85^a^	0.5206^ab^	2.00^b^	3.84	40.3933^c^	5.8933^b^	29.4200^b^
Sucralose	2.79^a^	0.5206^ab^	2.00^b^	3.84	40.4733^c^	5.8000^c^	29.2333^bc^
Stevia	2.80^a^	0.5262^a^	1.83^bc^	3.48	41.2267^b^	5.7867^c^	29.2133^c^
Ciclamate/saccharin blend 2:1	2.80^a^	0.5150^b^	1.67^c^	3.24	40.6700^c^	5.7867^c^	29.3433^bc^
Neotame	2.81^a^	0.5206^ab^	1.75^bc^	3.36	41.1300^b^	5.8167^c^	29.3433^bc^

Means with same letters in a same line each parameter indicate that samples do not have statistical difference at a significance level of 5% by Tukey's means test.

* L = luminosity; +a = red, −a = green; +b = yellow, −b = blue.

1 Ratio of °Brix and titratable acidity (%).

According to Etxeberria and Gonzalez (2005) and Cadena et al. (2013), since sucrose is a soluble solid, this showed a significant influence in relation to °Brix, with a higher value in this sample. Therefore, these samples (sweetened with sucrose), showed a much higher ratio than the other samples, due to the increase in soluble solids.

There were significant differences (*P* > 0.05) in the color parameters (L*, a*, b*) (Table3). The passion fruit juice samples that were lighter in color, the values for the parameter of luminosity (L*) retracting and the intensity of the yellow color (b*) were lower. The passion fruit juice samples that were darker in color, the values for the parameter of luminosity (L*) retracting and the intensity of the yellow color (b*) were higher, as in the case of the sample with sucrose. According to Brito and Bolini (2010) and Cadena et al. (2013), this darkened color could be associated with nonenzymatic processes with the formation of caramel colored pigments.

### Equi-sweetness determination

The ideal sweetness analysis revealed that 9.4/100 g was the ideal sucrose concentration. The relative sweetness analysis showed that neotame presented the highest sweetening power, being 6025.64 times sweeter than sucrose in relation to passion fruit juice containing 9.4/100 g of sucrose, followed by sucralose (590.02), cyclamate/saccharin blend 2:1 (262.28), aspartame (171.62), and stevia (94.72). These results were described according to Rocha and Bolini (2014).

### Sensory profile

Table4 shows the results of each sample for all the 18 descriptors generated by the trained panel.

**Table 4:** Attributes of the descriptive sensory evaluation by the trained panel for each passion fruit juice sample (*n* = 12 judges)

	Sucrose	Aspartame	Sucralose	Stevia	Cyclamate: saccharin blend 2:1	Neotame	MSD^*^
Yellow color	5.84^a^	5.62^a^	5.82^a^	5.65^a^	5.81^a^	5.58^a^	0.3852
Apparent viscosity	2.48^a^	2.22^a^	2.38^a^	2.24^a^	2.29^a^	2.28^a^	0.4077
Brightness	7.13ª	7.11ª	7.15ª	7.08ª	7.07ª	7.06^a^	0.3159
Passion fruit aroma	5.26ª	5.14ª	5.18ª	5.14ª	5.20ª	5.13ª	0.4892
Sweet aroma	2.99ª	3.08ª	3.11ª	3.04ª	2.98ª	3.26ª	0.4861
Acid aroma	3.84ª	3.65ª	3.69ª	3.78ª	3.73ª	3.72ª	0.5341
Cooked aroma	1.26ª	1.54ª	1.51ª	1.58ª	1.54ª	1.58ª	0.4452
Passion fruit flavor	5.25ª	5.12ª	5.18ª	4.67^b^	4.96ª^b^	5.03ª^b^	0.4322
Cooked flavor	1.50ª	1.54ª	1.41ª	1.65ª	1.77ª	1.50ª	0.5688
Sweet taste	3.80^c^	4.67^ab^	4.03^bc^	4.12^abc^	3.53^c^	4.80ª	0.7540
Bitter taste	0.46^c^	0.62^c^	0.87^c^	3.64ª	1.56^b^	0.97^c^	0.5842
Sweet aftertaste	1.05^de^	2.35^bc^	1.77 ^cd^	2.61^ab^	1.15 ^cd^	3.17ª	0.6815
Bitter aftertaste	0.33^c^	0.39^c^	0.64^c^	3.56^a^	1.39^b^	0.71^c^	0.5359
Sour taste	3.45^ab^	3.38^b^	3.74^ab^	3.53^ab^	3.74^ab^	3.95ª	0.5166
Sour aftertaste	2.50ª^b^	2.38^b^	2.69^ab^	2.60^ab^	2.80^ab^	2.94ª	0.5390
Adstringency	3.48^ab^	3.41^b^	3.64^ab^	3.64^ab^	3.65^ab^	3.94ª	0.5193
Metallic taste	0.43^b^	0.50^b^	0.52^b^	0.93ª	0.50^b^	0.52^b^	0.3077
Viscosity	2.19ª	2.14ª	2.27ª	2.23ª	2.23ª	2.13ª	0.3545

Means in the same line showing common letter are not significantly different (*P* = 0.05). MSD, minimum significant difference.

**Figure 1:**
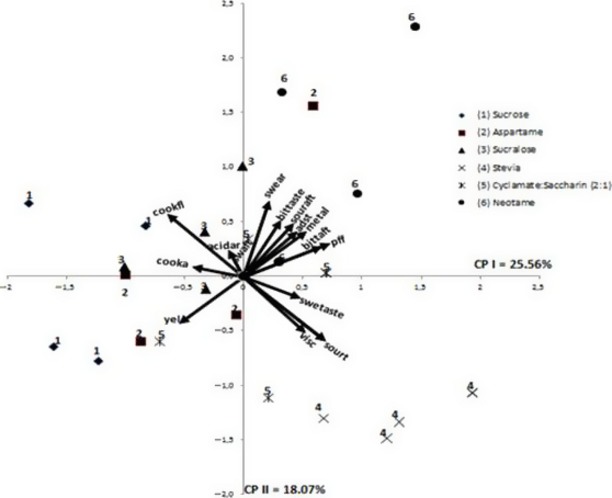
PCA generated with the sensory data for appearance, aroma, flavor, and texture.

Figure1 shows the results for the PCA to illustrate the similarities and differences amongst the passion fruit juice samples with respect to their attributes.

In Figure1, the sensory descriptors are represented as vectors and the passion fruit juice as numbers from 1 to 6. Each sample is represented 4 times, corresponding to the repetitions performed by the descriptive panel. The Principal Components I, II and III explain 55.7% of the total sensory variation amongst the samples. This percentage can be explained because no differences were observed in passion fruit juice with sucrose and sweeteners in many attributes.

As shown in Figure1, the position of the three samples sweetened with sucrose, aspartame, and sucralose suggests that these samples presented similar characteristics to each other.

In this study, no differences were observed in color and brightness of passion fruit juice samples with sucrose and sweeteners. These findings are consistent with samples sweetened with different sweeteners and sucrose in peach nectar (Cardoso and Bolini 2008), grape nectar (Voorpostel et al. 2014), and diabetic/reduced calorie chocolate (Melo et al. 2009).

No significant (*P* < 0.05) differences were found for all descriptive terms of the attribute aroma (passion fruit, cooked, acid and sweet), suggesting that those sweeteners had little influence on this attribute. Similar results were observed in a study on grape nectar (Voorpostel et al. 2014).

The presence of cooked aroma and cooked flavor may be due to the heat treatment of passion fruit juice (pasteurization), as described by Sandi et al. 2003.

The average scores for the attribute sweetness were significantly (*P* < 0.05) higher for neotame, aspartame, and stevia, differing in relation to cyclamate/saccharin 2:1 and sucrose, which exhibited the lowest scores for this attribute. The lower sweeteness observed in nectar sweetened with cyclamate/saccharin 2:1 was also reported in mango pulp by Umbelino (2005). Although this result could be inconsistent with those obtained in the sweetness equivalence test, as reported by Umbelino (2005), it may be due to the sweetness equivalence of sweet taste was assessed globally, while QDA assessed the evaluation of initial and residual sweetness separately.

Some undesirable descriptors were mentioned such as bitter taste, bitter aftertaste, sweet aftertaste, and metallic flavor, probably due to use of sweeteners (Brito and Bolini 2010).

Samples with stevia presented the most bitter taste, bitter aftertaste, and metallic flavor, and samples with neotame presented the sweetest aftertaste, as also reported by Cardoso and Bolini (2008). Furthermore, the sample with stevia presented lower scores for passion fruit flavor. The undesirable descriptors bitter taste, bitter aftertaste, and metallic flavor may have hindered the passion fruit flavor (Brito and Bolini 2010).

Samples sweetened with sucrose, aspartame, sucralose, and neotame did not present bitter taste or bitter aftertaste, and sweet aftertaste was not perceived in sucrose and sucralose samples either. These occurrences were reported in other studies on these sweeteness (Cardoso and Bolini 2008; Brito and Bolini 2010; Cadena et al. 2013).

Samples sweetened with aspartame showed intermediate intensity of sweet aftertaste associated with low intensity of sour taste, sour aftertaste, and adstringency. This characteristic can be due the sweetener presented a significantly higher intensity of fruit flavor than the sample sweetened with sucrose (Cloninger and Baldwin 1974). Similar result was reported by Cavallini and Bolini (2003) in mango juices, because aspartame elicited a significantly longer persistence of fruitiness, suggesting an intensification effect on fruitiness of mango juice. Therefore, the low intensity of sour taste, sour aftertaste, and adstringency may be due to the higher intensity of fruit flavor of aspartame.

Similar sensory profiles (*P* < 0.05) were observed for the samples with sucrose, aspartame, and sucralose, which did not exhibit bitter taste, bitter aftertaste, and metallic taste, with a higher intensity of the attribute passion fruit flavor; in addition, sucrose and sucralose presented similar results for sweet aftertaste (*P* < 0.05). Therefore, these results demonstrate that aspartame and sucralose are the best sucrose substitutes.

### Acceptance test

Amongst the consumers (*n* = 124), 67.74% were female and 32.26% were male. The participants were between 18 and 30 (90.32%) and 31 or more years old (9.68%). The volunteers were PhD students (38.71%) and graduated students (54.84%).

The 124 consumers evaluated the passion fruit juice samples for appearance, aroma, flavor, texture, and overall impression. The results are presented in Table5.

**Table 5:** Mean scores obtained by consumers (*n* = 124) in the acceptance test of passion fruit juice samples

	Sucrose	Aspartame	Sucralose	Stevia	Cyclamate/saccharin	Neotame	MSD
Appearance	6.37^a^	6.44^a^	6.29^a^	6.27^a^	6.19^a^	6.16^a^	0.3615
Aroma	5.79^ab^	6.07^a^	5.91^ab^	5.55^b^	5.87^ab^	5.83^ab^	0.5083
Flavor	5.79^a^	5.87^a^	5.77^a^	2.92^c^	4.69^b^	4.24^b^	0.6570
Texture	6.36^abc^	6.55^a^	6.40^ab^	5.53^d^	5.96 ^cd^	6.03^bc^	0.4412
Overall impression	6.09^a^	6.27^a^	5.98^a^	3.77^c^	5.28^b^	4.85^b^	0.5471

Means in the same line showing common letter are not significantly different (*P* = 0.05). MSD, minimum significant difference.

According to the results in Table5, there was no significant difference (*P* > 0.05) for the attribute appearance between the passion fruit juice samples evaluated by consumers. Similar results were observed in grape nectar (Voorpostel et al. 2014), diabetic/reduced calorie chocolates (Melo et al. 2009), concentrated reconstituted pineapple juice (Marcellini et al. 2005), guava nectar (Brito and Bolini 2008a), and acerola nectar (Dutra and Bolini 2013).

Regarding the attribute aroma, the samples with aspartame presented high scores, followed by sucralose and cyclamate/saccharin blend 2:1. Samples with aspartame only differed from the samples sweetened with stevia. However, Marcellini et al.(2005) found no difference among samples of reconstituted pineapple juice, while Brito and Bolini (2008a) reported that the samples of guava nectar sweetened with sucrose, sucralose, and aspartame had the highest scores for this attribute.

For the attributes flavor, texture, and overall impression, the sample with aspartame showed higher sensory acceptance, and it did not differ from the sample sweetened with sucralose and sucrose (*P* < 0.05). This characteristic may be due to the significantly higher intensity of fruit flavor of aspartame, thus it elicited significantly longer persistence of fruitiness (Cloninger and Baldwin 1974; Cavallini and Bolini 2003; Brito and Bolini 2008b).

Brito and Bolini (2008a) also reported that the samples of guava nectar with sucrose, sucralose, and aspartame had the highest flavor scores.

The sample sweetened with stevia had the lowest acceptance regarding the attributes aroma, flavor, texture, and overall impression (*P* < 0.05). Stevia presents undesirable descriptors for beverages such as bitter taste, bitter aftertaste, and metallic flavor (Table4). Several authors have reported these stevioside characteristics, including Voorpostel et al. (2014) (grape nectar), Melo et al. (2009) (chocolate), Dutra and Bolini (2013) (acerola nectar), and Fernandes et al. (2009) (guava nectar). According to these studies, the samples with stevia had lower acceptance for the attribute flavor. Therefore, the lowest acceptance scores may be due to these descriptors that almost covered the sweet taste starting at concentration equi-sweet to 20% sucrose, as also reported by Bolini-Cardello et al. (1999).

**Figure 2:**
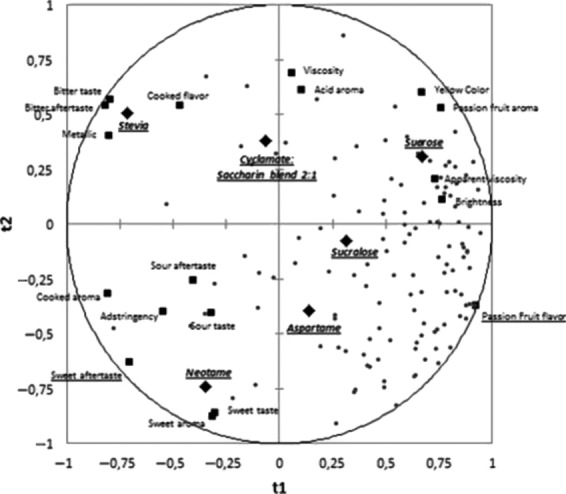
External preference map (X and Y are horizontal and vertical axes, respectively) obtained by partial least squares regression of descriptive data and respondent's overall liking scores for the sensory attributes of passion fruit juice (square = samples; circle = consumers; triangle = attributes of quantitative descriptive analysis).

**Figure 3:**
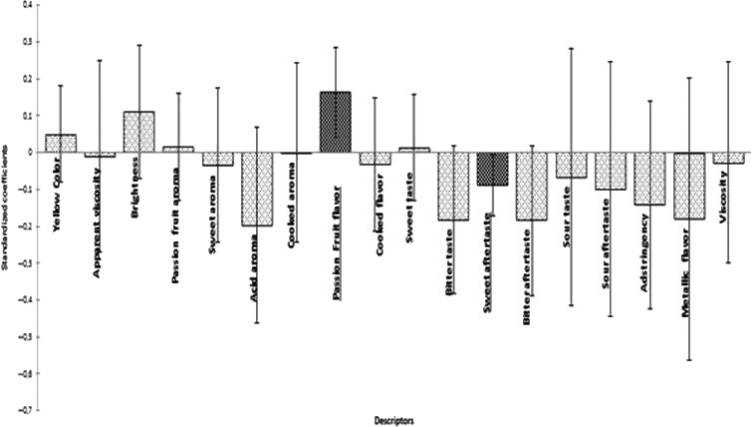
Partial least squares standardized coefficients of passion fruit juice (darker = descriptor terms without significant contribution to consumer acceptance).

The correlation between the overall impression and sensory descriptors data using PLS regression is shown in Figures2, 3. PLS is one of the modeling approach used when predictive variables are intercorrelated (Tang et al. 2000; Melo et al. 2009). PLS is a multivariate method suitable for the analysis of sensory descriptors and overall impression by consumers. Furthermore, it may be useful to guide the selection of a subset of relevant attributes from the complete set of attributes, and the number of significant components to be evaluated is usually determined by a cross-validation procedure (Rossini et al. 2012).

The purpose of PLS is to establish the sensory attributes that are mainly related to the preference of the passion fruit juice sweetened with different sweeteners, and to determine the attributes that have contributed positively and negatively to consumer's acceptance, verifying its degree of influence (Morais et al. 2014).

According to Figure2, the columns represent the sensory descriptors. Columns located on the positive portions of the Y axis are considered to be positively correlated with the acceptance of the passion fruit samples, while columns on the negative portion of the Y axis represent the attributes that were negatively correlated with the acceptance of the samples (Cadena et al. 2013; Gomes et al. 2014).

The column size represents the effect (positive or negative) of the attribute on the sample acceptance, and the vertical line represents the 95% confidence interval. It should be noted that when the vertical line crosses the X axis, the correspondent attribute does not have an influence on the drivers of preference (Gomes et al. 2014).

Furthermore, Figure2 shows that the attribute passion fruit flavor affected positively the acceptance of the passion fruit juice samples, while the attribute sweet aftertaste affected negatively. Despite a negative effect of sweet aftertaste was found in mango juice (Cadena et al. 2013), this attribute presented a positive effect on vanilla ice creams with reduced fat and sugar (Cadena et al. 2012). A positive effect of fruit flavor was found by Voorpostel et al. (2014) in grape nectar.

According to the External Preference Map (Fig.3), the consumers (circles) were close to the samples (squares) with the highest acceptance scores. Most of the consumer groups were near the passion fruit samples with sucrose, aspartame, and sucralose, which were characterized by the attribute passion fruit flavor, whose intensity may have influenced consumers' acceptance. The neotame was associated with sweet aftertaste, sour taste, sour aftertaste, and adstringency, and stevia was associated with bitter taste, bitter aftertaste, and metallic flavor, which are undesirable descriptors for this sample. Probably the association between these attributes and those that contributed to a better acceptance of the product may have influenced the lower mean scores observed for the samples with neotame and stevia, when compared with the other samples. These results demonstrate that the presence of some types of sweeteners can influence the preference of passion fruit juice by the consumers.

## Conclusion

According to QDA, the sweeteners aspartame and sucralose showed a sensory profile similar to sucrose, once the consumers that participated in the study preferred the samples sweetened with aspartame, sucralose, and sucrose, which received the highest scores for the attributes flavor, texture, and the overall impression.

These results have proved that aspartame and sucralose are the best sucrose substitutes, because these sweeteners presented a high intensity of passion fruit flavor, and did not present bitter taste, bitter aftertaste, and metallic taste.

The occurrence of undesirable descriptors (sweet aftertaste, bitter, bitter aftertaste, and metallic taste) is a constant problem when dealing with sweeteners. Thus, more studies are required for developing new sweeteners without these undesirable descriptors.
